# Extracorporeal Shockwave Therapy Improves Outcome after Primary Anterior Cruciate Ligament Reconstruction with Hamstring Tendons

**DOI:** 10.3390/jcm12103350

**Published:** 2023-05-09

**Authors:** Patrick Weninger, Christoph Thallinger, Manuel Chytilek, Yannis Hanel, Caterina Steffel, Ramin Karimi, Xaver Feichtinger

**Affiliations:** 1 Sports Medical Center, Am Hof 11/9, 1010 Vienna, Austria; christoph.thallinger@aon.at (C.T.); ordination@dr-weninger.at (M.C.);; 2 Döbling Private Clinic, Heiligenstädter Straße 55-63, 1190 Vienna, Austria

**Keywords:** anterior cruciate ligament, extracorporeal shock wave therapy, graft maturation

## Abstract

Purpose: The decision regarding the timepoint of a return to sports after anterior cruciate ligament (ACL) reconstruction is complex and depends on many factors, including objectively tested physical and psychological readiness as well as biological healing. The aim of this study was to investigate the influence of repetitive extracorporeal shockwave therapy (ESWT) on return-to-sports duration, clinical results and MRI results after ACL reconstruction with hamstring tendons (HT). Material and Methods: In this prospective controlled study, all patients with acute ACL ruptures were treated by ACL reconstruction with HT. Patients were randomized into two groups (Group A: ESWT group; Group B: control group). Patients in the ESWT group received focused shockwave therapy 4, 5 and 6 weeks after ACL surgery. Follow-up investigations including IKDC score, Lysholm score, VAS and evaluation regarding return-to-sports timepoints that were conducted 3-, 6-, 9- and 12-months post-operation. An MRI investigation was performed 12-months post-operation and graft maturation (signal intensity ratio (SIR)) as well as femoral and tibial tunnel characteristics (bone marrow oedema, tunnel fluid effusion) were assessed. Results: In total, 65 patients (27.65 ± 7.07 years; 35 male/30 female) were included in this study. The mean timepoint for “return-to-pivoting-sports” was 27.92 weeks (±2.99) in the ESWT group as well as 42.64 weeks (±5.18) in the control group (*p* < 0.001). In the ESWT group 31 patients (vs. control group: *n* = 6) attained the “pre-injury activity level”, whereas 6 patients (vs. control group: *n* = 22) did not reach this level within 12 months post-operation. The IKDC score, Lysholm score, and VAS showed significant improvement in the ESWT group compared with the control group for all time-points (*p* < 0.001). The mean SIR in the ESWT group revealed 1.81 (±0.88), whereas the control group showed a mean SIR of 2.68 (±1.04) (*p* < 0.01). Discussion: In conclusion, this is the first study investigating the effect of repetitive ESWT on ACL reconstruction with clinical outcome measurements, including the duration of return-to-sports activity and an MRI follow-up examination. Return-to-sports parameters, clinical scores and graft maturation were significantly improved in the ESWT group. This study may support an earlier return-to-sports timepoint by ESWT and is of high clinical relevance as ESWT is a cost-effective treatment option with no relevant side effects.

## 1. Introduction

Knee ligament injuries represent the most frequent injuries of the musculoskeletal system [1]. Injuries of the anterior cruciate ligament (ACL) are common among professional and recreational athletes [2,3]. Rupture of the ACL leads to knee instability and may cause secondary damage to knee structures such as menisci and/or cartilage [4,5]. Reconstruction of the torn ACL is a frequently performed surgical procedure to avoid instability of the injured knee joint. Reconstruction of the ACL with hamstring tendon (HT) grafts is the most frequently used surgical method worldwide in ACL reconstruction [6]. The “return-to-sports” decision after ACL reconstruction is a complex process and depends on many factors, such as objectively tested physical and psychological readiness as well as biological healing [7]. Earlier studies have described the detection of biological healing and graft maturation by magnetic resonance imaging (MRI) [8,9,10,11]. In a sheep model, lower revascularization and graft maturation were associated with lower mechanical function and reduced long-term stability [12]. It is commonly known that, after ACL reconstruction, the graft undergoes an avascular necrosis and revascularization process [13]. MRI studies following HT ACL reconstruction have shown ongoing and gradual remodelling and graft maturation up to 2 years after reconstruction [14]. Thus, monitoring of the remodelling process might be crucial to determine when unrestricted sports activities may resume after ACL reconstruction [15,16]. Early but also safe “return-to-sports” is important after ACL reconstruction to lower the risk of a second injury to the ACL graft.

Extracorporeal shockwave therapy (ESWT) has shown its influence on bone and soft tissue regeneration both in experimental as well as clinical studies [17,18]. It has become an extensively used treatment option for orthopaedic overuse and also acute injuries of tendons, muscles and the tendinous junction [19,20]. Increased blood flow, modulation of cell proliferation in tendon and muscle regeneration, inflammation regulation as well as its influence on bone metabolism seem to be its key mechanisms [18,21,22,23,24]. In most orthopaedic protocols, 1500 impulses are used [25]. Studies investigating the effect of ESWT on ligament regeneration and its benefits on healing mechanisms after ACL reconstruction are rare. Wang et al. have reported outcomes after ESWT following ACL reconstruction showing improved Lysholm scores, decreased tibial tunnel enlargement, and decreased antero-posterior laxity in comparison with a control group [24].

The aim of this study was to investigate the influence of repetitive ESWT on return-to-sports duration, clinical results and MRI results in comparison with a control group after ACL reconstruction but without a post-operative ESWT protocol.

We hypothesized that repetitive extracorporeal shockwave therapy improves the clinical outcome as well as graft maturation after ACL reconstruction.

## 2. Materials and Methods

This controlled prospective clinical study was approved by the local ethics committee (No: 15-127-0715). The study was conducted in line with the current version of the Declaration of Helsinki. Informed consent was obtained from all individual participants included in the study.

Inclusion criteria were defined as acute ACL rupture treated with HT autograft. Patients were included if they were professional athletes or hobby athletes (at least 3 sport events per week during mid-season). Exclusion criteria were pregnancy, patients who were suffering from diabetes or vascular diseases, and patients who had undergone immunosuppressive or corticosteroid therapy within 6 weeks before treatment.

Patients were included between February 2017 and August 2017. Preoperatively, patients were randomized into two groups (A: ESWT group; B: control group).

All surgeries were performed by the same experienced knee surgeon. The operative technique was conducted with the use of an HT autograft as a standard procedure in both study groups [26]. Graft harvesting of the gracilis and semitendinosus tendon is typically conducted. An 8- to 9-mm graft is then prepared with a baseball stitch with no. 2 FiberWire^®^ (Arthrex, Naples, FL, USA). Then, a TightRope^®^-RT (Arthrex) is loaded and pretensioning is performed. Additionally a vancomycin solution (5 mg/mL) for presoaking is applied [26]. Then, the femoral tunnel is addressed (between the anteromedial and posterolateral bundle insertion point) with drilling through the anteromedial portal at a 120° knee flexion. Subsequently, the tibial tunnel is prepared with an aiming device placed according to the anatomic footprint. Then, the prepared autograft is passed through the tunnels and the TightRope^®^-RT is flipped by standard. Tibial fixation is performed with an interference screw as a standard procedure [26].

All patients received the same post-operative rehabilitation protocol according to “Early Active Rehabilitation” [26]. Thereby, patients focus on immediate full weight-bearing without any bracing. With isokinetic muscle activation being performed from the first post-operative day and onward.

Post-operatively, patients of the ESWT group received shockwave therapy exactly 4, 5 and 6 weeks after surgery. For ESWT we used a standardized protocol once a week for these three weeks, wherein a total of 1500 impulses were applied. A total of 500 impulses were focused on the central part of the knee joint through the lateral soft spot over the lateral femorotibial space. A total of 500 impulses were focused on the femoral tunnel laterally and 500 impulses on the tibial tunnel by an electromagnetic generation mechanism with an energy flux density of 0.25 mJ/mm^2^ at 5 Hz (Duolith^®^ SD1 «ultra», Storz Medical AG, Tägerwilen, Switzerland). Patients belonging to the control group received no shockwave treatment.

Standard post-operative follow-up investigations were performed 3, 6, 9 and 12 months after operation by the same experienced knee surgeon blinded regarding the study group.

As the primary outcome parameter, the timepoint of “return-to-pivoting-sports” was evaluated. Furthermore, patients were asked questions regarding the timepoint of “return-to-running activity”, as well as “return to pre-injury activity level”.

Clinical evaluation was performed at 3-, 6-, 9- and 12-months post-surgery using VAS, the International Knee Documentation Committee (IKDC) score [27], and the Lysholm score [28]. A radiological evaluation was performed by using magnetic resonance imaging.

An MRI scan was performed twelve moths post-operatively.

**Figure 1:**
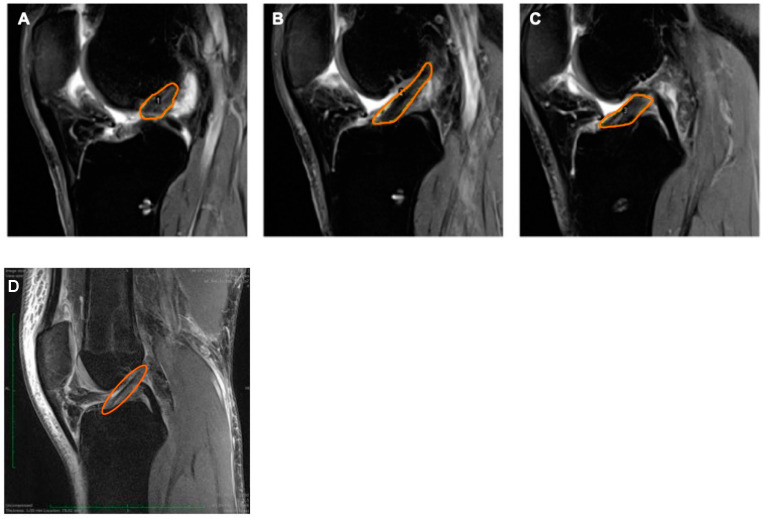
Sagittal MRI scans (T2 weighted sequence) with marked (orange) ACL as region of interest for signal intensity ratio calculations. (**A**) SIR lateral to the PCL, (**B**) SIR at level of PCL, (**C**) SIR medial to the PCL, and (**D**) intact (healthy) ACL.

All MRI scans were evaluated by the same investigator, who was blinded regarding the study group. T2-weighted MRI scans were used for assessment. The imaging program ImageJ (Version 1.52; National Institutes of Health, Bethesda, MD, USA) was used for quantification of the scans [29,30]. Thereby, a region of interest (ROI) was defined manually including the intra-articular part of the ACL graft for each slice and grey values were evaluated (0 (black)–255 (white)) (Figure 1). Subsequently a mean grey value (MGV) was created for each ROI to compare values statistically.

The signal intensity ratio (SIR) was used to assess the grey values of the graft, the ratio of MGV of the ACL (Figure 1) of the posterior cruciate ligament (PCL) in sagittal slices.

**Figure 2:**
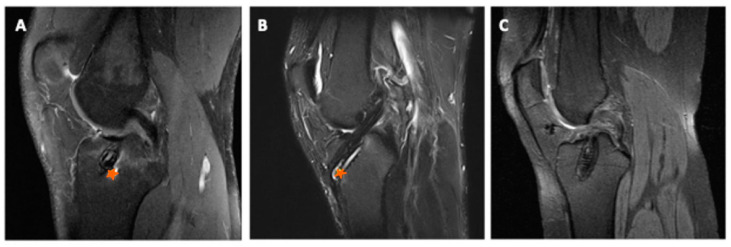
Different sagittal MRI scans showing bone marrow oedema ((**A**), orange star), tunnel liquid effusion ((**B**), orange star) and no signs of oedema or liquid effusion (**C**).

The femoral and the tibial tunnel were evaluated for tunnel widening and fluid within the tunnels and for the presence of tunnel liquid effusion and tunnel walls bone marrow oedema. According to earlier studies, liquid within the femoral or the tibial tunnel was determined via the presence of a hyperintense signal band or cyst (absence: 0; presence: 1). Tunnel walls bone marrow oedema was assessed by evaluating the area in the cancellous bone around the graft for a hyperintense signal (absence: 1; presence: 0) [31] (Figure 2).

### Statistical Analysis

For statistical analysis, GraphPad Prism version 8.3.1 (GraphPad Software, La Jolla, CA, USA, www.graphpad.com) and SPSS version 26 (IBM Corp., Armonk, NY, USA) were used. Based on a clinical study by Wang et al. [24], which evaluated clinical and radiographic outcome after ACL reconstruction with and without ESWT and assuming a power of 0.80 and a significance level of 5%, at least 27 patients are required for statistical analyses. Testing for normal distribution was performed using the Shapiro–Wilk test. In case of a normal distribution, unpaired t-tests were used for analyses. If there was no normal distribution, the non-parametric Mann–Whitney U test was used. A *p* value of <0.05 was considered statistically significant.

## 3. Results

In total, 65 patients (mean ± standard deviation; 27.65 ± 7.07 years; 35 male/30 female) were included in this study. The patient cohort was randomized into the study groups as follows: ESWT group (37 patients, mean 28.51 ± 7.42 years); control group (28 patients, mean 26.50 ± 6.52 years). All patients took part in return-to-activity evaluation as well as clinical evaluation. The two most often performed sports were alpine skiing (26.2%) and football (21.5%). The time between injury and surgery was without significant differences between the two groups (ESWT group: mean 7.49 weeks; control group: mean 7.46 weeks).

A total of 49 patients (mean 27.43 ± 6.99 years; 30 male/19 female) were available for radiographic evaluation 12 months (±4 weeks) post-operatively. A total of 16 patients were not available for MRI analyses due to scheduling reasons, lack of motivation, claustrophobia, T2-weighted slices not available, etc.

### 3.1. Return-to-Activity Evaluation

The mean timepoint for “return-to-pivoting-sports” was 27.92 weeks (±2.99) in the ESWT group and 42.64 weeks (±5.18) in the control group (*p* < 0.001). “Return-to-running activity” was 10.46 weeks (±1.48) in the ESWT group and 18.46 weeks (±3.28) in the control group (*p* < 0.001).

In the ESWT group 31 patients attained the “pre-injury activity level”, whereas 6 patients did not reach this level within 12 months post-operation. In the control group, 6 patients reached their “pre-injury activity level”, whereas 22 patients were not able to perform according to their pre-operative activity level within the follow-up investigations (*p* < 0.001).

### 3.2. Clinical Scores

**Figure 3:**
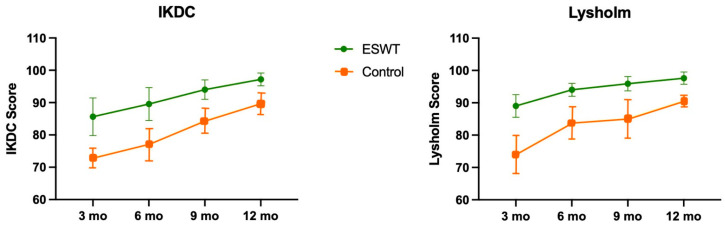
International Knee Documentation Committee (IKDC) (**left**) and Lysholm score (**right**) with mean values and standard deviation for different time points for both study groups (ESWT group: green; control group: orange).

**Figure 4:**
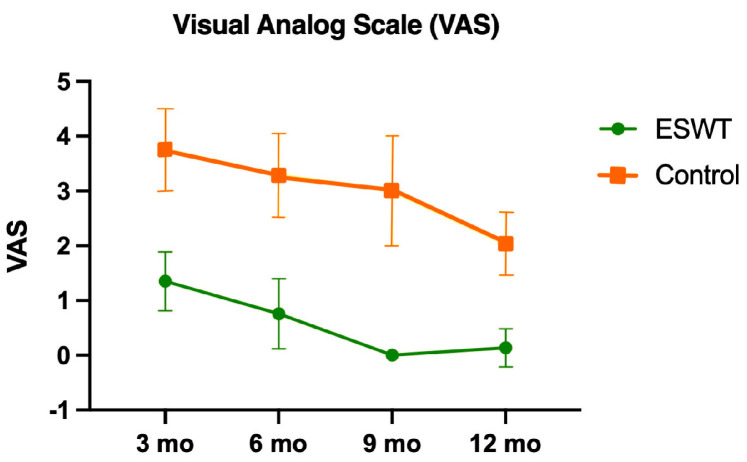
Visual analogue scale (VAS) with mean values and standard deviation for different time points for both study groups (ESWT group: green; control group: orange).

The IKDC score as well as the Lysholm score showed significant improvement (*p* < 0.001) in the ESWT group compared with the control group for all time-points (3, 6, 9, 12 months) (Figure 3). The VAS showed less pain (*p* < 0.001) in the ESWT group for all time points (3, 6, 9, 12 months) (Figure 4).

### 3.3. Radiographic Evaluation

**Figure 5:**
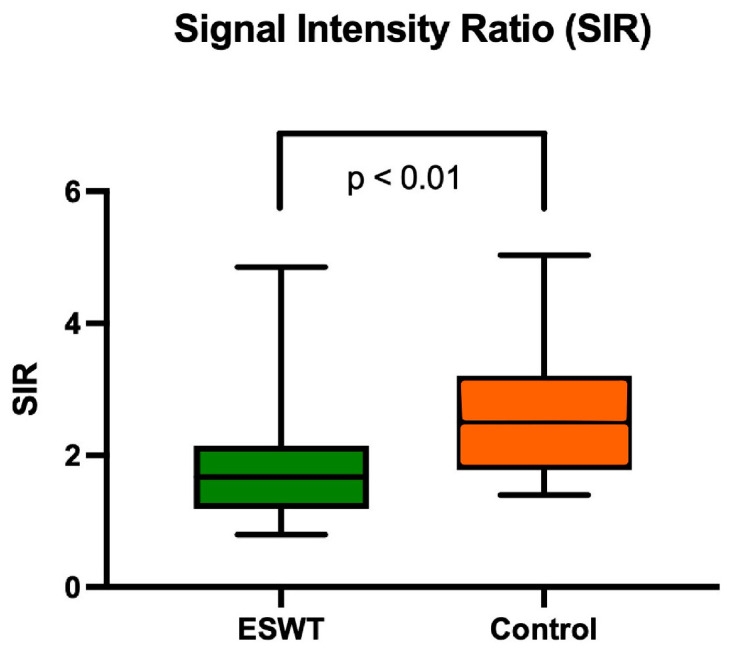
Signal intensity ratio (SIR) for both study groups 12 months after ACL reconstruction. Values are presented as median, interquartile range and minimum/maximum.

SIR evaluation was available in 38 patients (ESWT group: 22; control group: 16) as sagittal T2-weighted images were not available in 11 patients. The mean SIR in the ESWT group revealed 1.81 (±0.88), whereas the control group showed a mean SIR of 2.68 (±1.04) (*p* < 0.01) (Figure 5).

Femoral and tibial tunnel evaluations were available in 45 patients (ESWT group: 27; control group: 18) as sagittal or frontal T2-weighted images were not available in 4 patients. In none of the patients were femoral tunnel walls bone marrow oedema detectable. Tunnel liquid effusion was seen in 2 patients in the ESWT group and in 4 patients in the control group (*p* < 0.05).

In the tibia, tunnel liquid effusion did not differ between the study groups (4 patients of the ESWT group and 5 patients of the control group). Tunnel walls bone marrow oedemas were seen in 3 patients in the ESWT group and in 4 patients in the control group (n. s.).

## 4. Discussion

This study investigated the influence of ESWT on the clinical and radiographic outcome of ACL reconstruction with HT. It was shown that the timepoint of “return-to-pivoting-sports”, as well as “return-to-running activity” was significantly shortened. Furthermore, the number of patients achieving their “pre-injury activity level” was significantly higher in the ESWT group. The clinical scores IKDC and Lysholm showed clearly improved rates in the ESWT group.

Radiographic analyses showed improved SIR in the ESWT group and less fluid effusion in the femoral tunnel. Bone marrow oedema, as well as fluid effusion in the tibial tunnel did not differ significantly between the two groups.

The effect of ESWT on bone and soft tissue regeneration has been described earlier both in experimental and clinical studies. Increased blood flow, modulation of cell proliferation in tendon and muscle regeneration, inflammation regulation as well as influence on bone metabolism seem to be key mechanisms [18,21,22,23,24,32]. Literature concerning ESWT in ACL reconstruction surgery is rare. Wang et al. investigated the influence of ESWT on bone–tendon interface in ACL reconstruction experimentally [33]. They were able to show significant improvement in bone–tendon interface healing in rabbits with increased trabecular bone around the tendon in the shockwave group [33]. Wang et al. later described the effect of one-off ESWT directly after surgery on ACL reconstruction with an improved Lysholm score, decreased tibial tunnel enlargement as well as decreased antero-posterior laxity in comparison with a control group [24]. However, they did not focus on the influence of ESWT on the timepoint of return-to-sports activity. Contrary to that study, the shockwave treatment in the present study was applicated repetitively 4, 5 and 6 weeks after surgery. Improvements in the duration of time by which pre-injury levels were attained were shown in this study population.

In the present study, the authors used the term “graft maturation” as an MRI-based parameter. This means that SIR values give an impression of the grey values that can be a sign for “ligamentization” during the graft remodelling process: the darker the graft, the more “mature” it is. This refers to vascularization and collagen type of the tendon graft during its ligamentization process [15,16].

Earlier studies have already investigated graft maturation of the ACL autograft. An MRI following HT ACL reconstruction showed ongoing gradual remodelling and graft maturation up to 2 years after reconstruction [14]. Li et al. were able to show that the graft bending angle of the autograft influences the graft maturation but has no effect on the clinical outcome after 12 months [34]. Contrary to that, Scheffler et al. associated—in an experimental study investigating sheep—delayed remodelling of the ACL with reduced long-term stability and mechanical function [12]. Van Dyck et al. described the heterogeneity of graft maturity measurement techniques and their poor correlation to clinical outcomes [35]. The present study showed improved clinical outcome as well as improved graft maturation—measured by SIR—in the ESWT treated group. The measurement techniques of graft ligamentization and the association with clinical outcome has to be studied in further prospective randomized trials.

To assess bone-tunnel healing after ACL reconstruction, important factors, such as bone-tunnel enlargement, liquid effusion in the bone tunnel, and bone marrow oedema around the tunnel, were described [31]. Earlier studies have described bone-tunnel healing as an important factor after ACL reconstruction, with a supposed effect on graft survival [36]. The present study showed less liquid effusion in the femoral tunnel in the ESWT group. These results may influence the ligamentization of the ACL graft and also might have an impact on tunnel widening. Tunnel wall bone marrow oedema and fluid effusion in the tibial tunnel did not differ between the groups.

### Strengths and Limitations

The present study has several strengths and limitations. One strength is the high number of patients taking part and the low number of patients who failed to follow up. Furthermore, the combination of imaging results in combination with clinical results and return-to-sports activity statements are of high clinical relevance as return to sports is a significant factor after ACL reconstruction. The time between injury and surgery does not differ significantly between the two groups and supports comparability between the two study groups.

One of the main and major limitations of the present study is the absence of a placebo group as control group. Only by using a placebo group is the role of ESWT as a single variable affecting the outcome parameters possible. At the moment, a follow-up study is being conducted using this placebo group with a double-blinded protocol. Furthermore, it needs to be pointed out that the role of MRI studies needs to be questioned due to its limited sensitivity and specificity for ACL graft maturation. The variability of MRI investigations with different scan quality due to the heterogeneity of the MRI institutions may influence the radiographic results. Further, inter-observer reliability might be a confounder when performing such studies. Therefore, this also needs to be highlighted as a study limitation. Another limitation is that no trend in SIR measurements was detectable due to the use of only a single MRI investigation per patient 12 months post-operation.

Although the present study shows trends that support ESWT after ACL reconstruction, further research is needed to investigate the long-term effects of ESWT on ACL reconstruction surgery.

## 5. Conclusions

In conclusion, this is the first study investigating the effect of repetitive ESWT on ACL reconstruction with clinical outcome measurements, including the duration of return-to-sports activity and an MRI follow-up investigation. It was shown that time to “return-to-pivoting-sports”, as well as “return-to-running activity” was significantly shortened in the ESWT group. The number of patients achieving their “pre-injury activity level” was significantly higher in the ESWT group. Clinical scores (IKDC, Lysholm, VAS) were significantly improved in the ESWT group for all timepoints and the graft maturation was clearly increased by ESWT. These results may support an earlier return-to-sports timepoint by ESWT and are of high clinical relevance as ESWT is a cost-effective treatment option with no relevant side effects.

Again, the limitations of the study need to be considered as described above. Although the present study shows trends that support ESWT after ACL reconstruction, further research is needed to investigate the long-term effects of ESWT on ACL reconstruction surgery.

## Data Availability

Data are stored at Sports Medical Center and can be requested there.
